# Ethyl 4-(2-bromo-5-fluoro­phen­yl)-6-methyl-1-phenyl-2-thioxo-1,2,3,4-tetra­hydro­pyrimidine-5-carboxyl­ate

**DOI:** 10.1107/S1600536808021685

**Published:** 2008-07-19

**Authors:** Hoong-Kun Fun, Samuel Robinson Jebas, M. Babu, P. S. Patil, B. Kalluraya, S. M. Dharmaprakash

**Affiliations:** a X-ray Crystallography Unit, School of Physics, Universiti Sains Malaysia, 11800 USM, Penang, Malaysia; b Department of Studies in Chemistry, Mangalore University, Mangalagangotri, Mangalore 574 199, India; c Department of Studies in Physics, Mangalore University, Mangalagangotri, Mangalore 574 199, India

## Abstract

In the title mol­ecule, C_20_H_18_BrFN_2_O_2_S, the pyrimidine ring adopts a flattened envelope conformation. The halogenated benzene ring is orthogonal to the planar part of the pyrimidine ring [dihedral angle = 89.05 (4)°], while the other phenyl ring is oriented at an angle of 85.14 (5)°. The ethoxy group is disordered over two orientations with site occpancies of *ca* 0.869 (4) and 0.131 (4). Intra­molecular C—H⋯Br and C—H⋯O hydrogen bonds generate *S*(5) and *S*(6) ring motifs. The crystal structure is stabilized by inter­molecular N—H⋯S, C—H⋯F, C—H⋯O and C—H⋯Br hydrogen bonds.

## Related literature

For the biological activity of pyrimidinone derivatives, see: Atwal (1990 ▶); Matsuda & Hirao (1965 ▶); Sadanandam *et al.* (1992 ▶). For the synthetic procedure, see: Steele *et al.* (1998 ▶); Manjual *et al.* (2004 ▶); Kappe (1993 ▶); Wipf & Cunningham (1995 ▶). For bond-length data, see: Allen *et al.* (1987 ▶). For ring conformations, see: Cremer & Pople (1975 ▶). For graph-set analysis of hydrogen bonding, see: Bernstein *et al.* (1995 ▶).
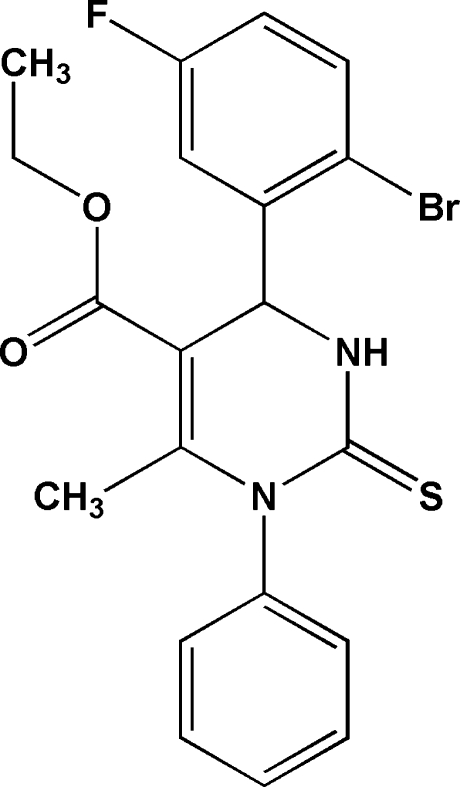


## Experimental

### 

#### Crystal data

C_20_H_18_BrFN_2_O_2_S
*M* *_r_* = 449.33
Triclinic, 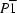

*a* = 10.0455 (1) Å
*b* = 10.2969 (1) Å
*c* = 10.3714 (1) Å
α = 64.286 (1)°
β = 83.110 (1)°
γ = 78.796 (1)°
*V* = 947.36 (2) Å^3^
*Z* = 2
Mo *K*α radiation
μ = 2.31 mm^−1^
*T* = 100 (2) K
0.41 × 0.35 × 0.22 mm

#### Data collection

Bruker SMART APEXII CCD area-detector diffractometer
Absorption correction: multi-scan (*SADABS*; Bruker, 2005 ▶) *T*_min_ = 0.451, *T*_max_ = 0.631
30132 measured reflections
5490 independent reflections
4895 reflections with *I* > 2σ(*I*)
*R*_int_ = 0.029

#### Refinement

*R*[*F* ^2^ > 2σ(*F* ^2^)] = 0.031
*wR*(*F* ^2^) = 0.084
*S* = 1.04
5490 reflections
267 parameters
15 restraints
H-atom parameters constrained
Δρ_max_ = 1.01 e Å^−3^
Δρ_min_ = −0.66 e Å^−3^

### 

Data collection: *APEX2* (Bruker, 2005 ▶); cell refinement: *APEX2*; data reduction: *SAINT* (Bruker, 2005 ▶); program(s) used to solve structure: *SHELXTL* (Sheldrick, 2008 ▶); program(s) used to refine structure: *SHELXTL*; molecular graphics: *SHELXTL*; software used to prepare material for publication: *SHELXTL* and *PLATON* (Spek, 2003 ▶).

## Supplementary Material

Crystal structure: contains datablocks global, I. DOI: 10.1107/S1600536808021685/ci2628sup1.cif

Structure factors: contains datablocks I. DOI: 10.1107/S1600536808021685/ci2628Isup2.hkl

Additional supplementary materials:  crystallographic information; 3D view; checkCIF report

## Figures and Tables

**Table 1:** Hydrogen-bond geometry (Å, °)

*D*—H⋯*A*	*D*—H	H⋯*A*	*D*⋯*A*	*D*—H⋯*A*
N1—H1*N*1⋯S1^i^	0.85	2.51	3.327 (2)	162
C1—H1⋯F1^ii^	0.95	2.52	3.370 (2)	148
C7—H7⋯Br1	1.00	2.69	3.265 (2)	117
C20—H20⋯O1^iii^	0.95	2.44	3.368 (3)	164
C21—H21*A*⋯O2	0.98	2.11	2.737 (3)	120
C21—H21*B*⋯Br1^iii^	0.98	2.91	3.886 (2)	171

Symmetry codes: (i) 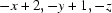; (ii) 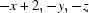; (iii) 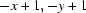, 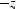.

**Fig. 1.**
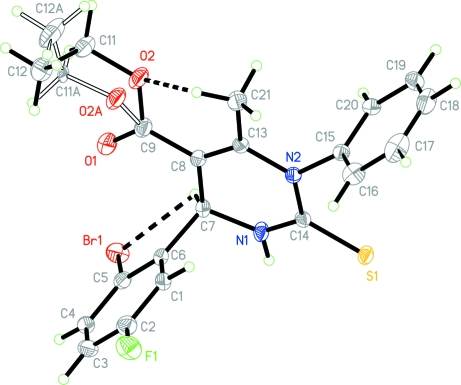
The molecular structure of the title compound, showing 50% probability displacement ellipsoids and the atom-numbering scheme. Both disorder components are shown.

**Fig. 2.**
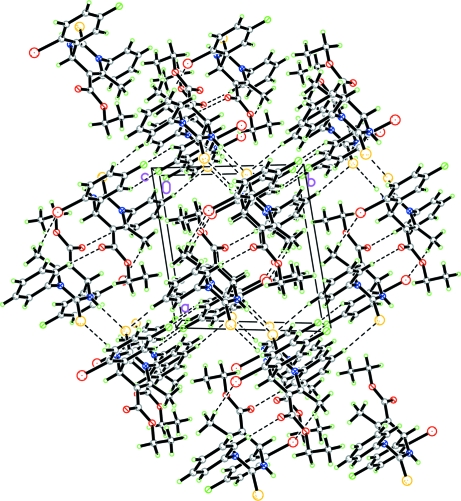
The crystal packing of the title compound, viewed along the c axis. Hydrogen bonds are shown as dashed lines. Only the major disorder component is shown.

## supplementary crystallographic information

### Comment

3,4-Dihydropyrimidinones have drawn wide-spread attention due to their pharmaceutical applications. A variety of these derivatives have been screened for antihypertension (Atwal, 1990), antibacterial (Matsuda & Hirao, 1965) and anti-inflammatory activities (Sadanandam *et al.*, 1992). The common synthetic routes to these compounds generally involve multi step transformations, which are essentially based on the Biginelli condensation methodology (Steele *et al.*, 1998). These pyrimidinones are also associated with calcium channel blocking activity (Manjual *et al.*, 2004). In 1893, Biginelli reported the first synthesis of dihydropyrimidines by a simple one-pot condensation reaction of ethyl acetoacetate, benzaldehyde and urea. In the following decades the original *cyclo*-condensation reaction has been extended widely to include variations in all three components, allowing access to a large number of muti functionalized dihydropyrimidinone derivatives (Kappe, 1993). Biginelli reaction has recently attracted a great deal of attention and several improved procedures for the preparation of dihydropyrimidinones have been reported within the past few years. Several solid-phase modifications of the Biginelli reaction suitable for the combinatorial chemistry have also been described (Wipf & Cunningham, 1995).

Bond lengths and angles in the title molecule (Fig. 1) are found to have normal values (Allen *et al.*, 1987). The pyrimidine ring adopts a flattened envelope conformation, with puckering parameters (Cremer & Pople, 1975) Q = 0.067 (2) Å, θ = 132.5 (16)° and φ = 237 (2)°. The C1-C6 and C15-C20 phenyl rings form dihedral angles of 89.05 (4)° and 85.14 (5)°, respectively, with the N1/N2/C7/C8/C13/C14 plane. Intramolecular C—H···Br and C—H···O hydrogen bonds generate S(5) and S(6) ring motifs (Bernstein *et al.*, 1995), respectively.

The crystal structure is stabilized by intermolecular N—H···S, C—H···F, C—H···O and C—H···Br hydrogen bonds (Table 1 and Fig.2).

### Experimental

A mixture of 2-bromo-5-fluorobenzaldehyde (0.01 mol, 2.0301 g), ethyl acetoacetate [0.015 mol, 2 g (2 ml)], phenylthiourea (0.01 mol, 1.5215 g) and concentrated H_2_SO_4_ (2 drops) in absolute alcohol (10 ml) taken in a beaker (100 ml) was put inside a microwave oven for 4 minutes at 160 Watts (25% MW power). The reaction mixture was then allowed to stand at room temperature and the product formed was filtered, washed with ethanol followed by water and dried. Further purification was done by recrystallization from ethanol (yield = 77%, m.p = 442–445 K). Composition calculated (found): C 53.45 (53.34), H 4.008 (3.92), N 6.236 (6.15), S 7.1269 (7.03)%.

### Refinement

The ethylcarboxylate group is disordered over two orienatations with refined occupancies of 0.869 (4):0.131 (4). The displacement parameters of atoms C11A and C12A were restrained to an approximate isotropic behaviour. The corresponding C—O and C—C distances in the two disorder components were restrained to be equal. All H atoms were positioned geometrically [C-H = 0.95–1.00 Å and N-H = 0.85 Å] and refined using a riding model, with *U*_iso_(H) = 1.2*U*_eq_(C,N) and 1.5_eq_(C_methyl_).

### Crystal data

**Table:**

C_20_H_18_BrFN_2_O_2_S	*Z* = 2
*M**_r_* = 449.33	*F*_000_ = 456
Triclinic, *P*1	*D*_x_ = 1.575 Mg m^−^^3^
Hall symbol: -P 1	Mo *K*α radiation λ = 0.71073 Å
*a* = 10.0455 (1) Å	Cell parameters from 9996 reflections
*b* = 10.2969 (1) Å	θ = 2.2–37.5º
*c* = 10.3714 (1) Å	µ = 2.31 mm^−^^1^
α = 64.286 (1)º	*T* = 100 (2) K
β = 83.110 (1)º	Block, colourless
γ = 78.796 (1)º	0.41 × 0.35 × 0.22 mm
*V* = 947.36 (2) Å^3^	

### Data collection

**Table:**

Bruker SMART APEXII CCD area-detector diffractometer	5490 independent reflections
Radiation source: fine-focus sealed tube	4895 reflections with *I* > 2σ(*I*)
Monochromator: graphite	*R*_int_ = 0.029
*T* = 100.0(1) K	θ_max_ = 30.0º
φ and ω scans	θ_min_ = 2.1º
Absorption correction: multi-scan(SADABS; Bruker, 2005)	*h* = −14→14
*T*_min_ = 0.451, *T*_max_ = 0.631	*k* = −14→14
30132 measured reflections	*l* = −14→14

### Refinement

**Table:**

Refinement on *F*^2^	Secondary atom site location: difference Fourier map
Least-squares matrix: full	Hydrogen site location: inferred from neighbouring sites
*R*[*F*^2^ > 2σ(*F*^2^)] = 0.031	H-atom parameters constrained
*wR*(*F*^2^) = 0.084	*w* = 1/[σ^2^(*F*_o_^2^) + (0.0443*P*)^2^ + 0.6872*P*] where *P* = (*F*_o_^2^ + 2*F*_c_^2^)/3
*S* = 1.05	(Δ/σ)_max_ = 0.001
5490 reflections	Δρ_max_ = 1.01 e Å^−^^3^
267 parameters	Δρ_min_ = −0.66 e Å^−^^3^
15 restraints	Extinction correction: none
Primary atom site location: structure-invariant direct methods	

### Special details

**Table:**

Experimental. The data was collected with the Oxford Cyrosystem Cobra low-temperature attachment.
Geometry. All e.s.d.'s (except the e.s.d. in the dihedral angle between two l.s. planes) are estimated using the full covariance matrix. The cell e.s.d.'s are taken into account individually in the estimation of e.s.d.'s in distances, angles and torsion angles; correlations between e.s.d.'s in cell parameters are only used when they are defined by crystal symmetry. An approximate (isotropic) treatment of cell e.s.d.'s is used for estimating e.s.d.'s involving l.s. planes.
Refinement. Refinement of *F*^2^ against ALL reflections. The weighted *R*-factor *wR* and goodness of fit *S* are based on *F*^2^, conventional *R*-factors *R* are based on *F*, with *F* set to zero for negative *F*^2^. The threshold expression of *F*^2^ > σ(*F*^2^) is used only for calculating *R*-factors(gt) *etc*. and is not relevant to the choice of reflections for refinement. *R*-factors based on *F*^2^ are statistically about twice as large as those based on *F*, and *R*- factors based on ALL data will be even larger.

### Fractional atomic coordinates and isotropic or equivalent isotropic displacement parameters (Å^2^)

**Table:**

	*x*	*y*	*z*	*U*_iso_*/*U*_eq_	Occ. (<1)
Br1	0.718042 (18)	0.65172 (2)	−0.42386 (2)	0.02506 (6)	
S1	0.96029 (4)	0.37018 (5)	0.22541 (4)	0.01943 (9)	
F1	1.01209 (13)	0.03875 (13)	−0.17700 (14)	0.0324 (3)	
O1	0.50168 (14)	0.40666 (18)	−0.20152 (16)	0.0313 (3)	
N1	0.81604 (15)	0.43472 (17)	0.00399 (15)	0.0196 (3)	
H1N1	0.8781	0.4859	−0.0370	0.023*	
N2	0.73979 (14)	0.26404 (16)	0.21844 (15)	0.0163 (3)	
C1	0.87086 (17)	0.2259 (2)	−0.13630 (19)	0.0209 (3)	
H1	0.8684	0.1647	−0.0365	0.025*	
C2	0.94486 (18)	0.1774 (2)	−0.2313 (2)	0.0236 (3)	
C3	0.95266 (18)	0.2600 (2)	−0.3770 (2)	0.0231 (3)	
H3	1.0052	0.2215	−0.4387	0.028*	
C4	0.88166 (17)	0.4003 (2)	−0.43062 (19)	0.0204 (3)	
H4	0.8841	0.4596	−0.5308	0.024*	
C5	0.80672 (16)	0.45529 (19)	−0.33865 (19)	0.0184 (3)	
C6	0.79770 (16)	0.37081 (19)	−0.19184 (18)	0.0175 (3)	
C7	0.71816 (17)	0.42662 (19)	−0.08487 (18)	0.0176 (3)	
H7	0.6680	0.5269	−0.1394	0.021*	
C8	0.61745 (16)	0.32950 (19)	0.00875 (19)	0.0182 (3)	
C9	0.50415 (18)	0.3362 (2)	−0.0741 (2)	0.0221 (3)	
C13	0.63308 (16)	0.24993 (19)	0.15063 (19)	0.0178 (3)	
C14	0.83214 (16)	0.35640 (18)	0.14314 (17)	0.0158 (3)	
C15	0.73718 (16)	0.19955 (18)	0.37368 (17)	0.0165 (3)	
C16	0.81160 (19)	0.0635 (2)	0.4482 (2)	0.0250 (4)	
H16	0.8712	0.0153	0.3985	0.030*	
C17	0.7980 (2)	−0.0018 (2)	0.5970 (2)	0.0339 (5)	
H17	0.8481	−0.0955	0.6498	0.041*	
C18	0.7111 (2)	0.0701 (3)	0.6680 (2)	0.0335 (5)	
H18	0.7008	0.0245	0.7694	0.040*	
C19	0.6396 (2)	0.2069 (3)	0.5931 (2)	0.0290 (4)	
H19	0.5817	0.2561	0.6429	0.035*	
C20	0.65228 (17)	0.2732 (2)	0.44396 (19)	0.0206 (3)	
H20	0.6033	0.3676	0.3914	0.025*	
C21	0.5474 (2)	0.1379 (2)	0.2489 (2)	0.0261 (4)	
H21A	0.5000	0.1072	0.1922	0.039*	
H21B	0.4807	0.1805	0.3027	0.039*	
H21C	0.6057	0.0531	0.3159	0.039*	
O2	0.40143 (16)	0.26503 (19)	0.00591 (18)	0.0270 (4)	0.869 (4)
C11	0.2885 (2)	0.2674 (3)	−0.0718 (3)	0.0290 (5)	0.869 (4)
H11A	0.2726	0.3622	−0.1566	0.035*	0.869 (4)
H11B	0.2050	0.2570	−0.0093	0.035*	0.869 (4)
C12	0.3186 (3)	0.1458 (3)	−0.1191 (3)	0.0319 (5)	0.869 (4)
H12A	0.2419	0.1492	−0.1712	0.048*	0.869 (4)
H12B	0.3329	0.0519	−0.0350	0.048*	0.869 (4)
H12C	0.4006	0.1568	−0.1819	0.048*	0.869 (4)
O2A	0.4326 (11)	0.2257 (8)	−0.0408 (13)	0.0270 (4)	0.131 (4)
C11A	0.3310 (13)	0.2655 (15)	−0.1426 (13)	0.018 (3)	0.131 (4)
H11C	0.3703	0.2613	−0.2331	0.021*	0.131 (4)
H11D	0.2784	0.3638	−0.1632	0.021*	0.131 (4)
C12A	0.247 (2)	0.147 (2)	−0.060 (2)	0.047 (5)	0.131 (4)
H12D	0.1690	0.1610	−0.1156	0.071*	0.131 (4)
H12E	0.2146	0.1512	0.0314	0.071*	0.131 (4)
H12F	0.3024	0.0519	−0.0417	0.071*	0.131 (4)

### Atomic displacement parameters (Å^2^)

**Table:**

	*U*^11^	*U*^22^	*U*^33^	*U*^12^	*U*^13^	*U*^23^
Br1	0.02414 (10)	0.02346 (10)	0.02129 (10)	0.00017 (6)	0.00032 (6)	−0.00558 (7)
S1	0.02016 (19)	0.0262 (2)	0.01355 (19)	−0.01177 (15)	0.00010 (14)	−0.00673 (16)
F1	0.0345 (6)	0.0264 (6)	0.0341 (7)	0.0048 (5)	−0.0013 (5)	−0.0146 (5)
O1	0.0241 (7)	0.0465 (9)	0.0253 (7)	−0.0044 (6)	−0.0057 (5)	−0.0162 (7)
N1	0.0233 (7)	0.0245 (7)	0.0142 (7)	−0.0131 (6)	0.0005 (5)	−0.0076 (6)
N2	0.0161 (6)	0.0205 (7)	0.0137 (6)	−0.0080 (5)	0.0022 (5)	−0.0072 (5)
C1	0.0198 (8)	0.0302 (9)	0.0189 (8)	−0.0095 (7)	0.0022 (6)	−0.0144 (7)
C2	0.0215 (8)	0.0237 (9)	0.0272 (9)	−0.0014 (6)	−0.0021 (7)	−0.0127 (8)
C3	0.0197 (8)	0.0279 (9)	0.0271 (9)	−0.0068 (7)	0.0052 (6)	−0.0167 (8)
C4	0.0186 (7)	0.0270 (9)	0.0190 (8)	−0.0086 (6)	0.0022 (6)	−0.0116 (7)
C5	0.0160 (7)	0.0203 (8)	0.0194 (8)	−0.0038 (6)	−0.0017 (6)	−0.0083 (7)
C6	0.0155 (7)	0.0232 (8)	0.0177 (8)	−0.0049 (6)	−0.0016 (5)	−0.0111 (7)
C7	0.0185 (7)	0.0219 (8)	0.0145 (7)	−0.0044 (6)	−0.0018 (5)	−0.0090 (6)
C8	0.0160 (7)	0.0229 (8)	0.0204 (8)	−0.0053 (6)	0.0006 (6)	−0.0130 (7)
C9	0.0187 (8)	0.0247 (9)	0.0289 (9)	−0.0017 (6)	−0.0039 (6)	−0.0167 (8)
C13	0.0155 (7)	0.0221 (8)	0.0205 (8)	−0.0068 (6)	0.0028 (6)	−0.0127 (7)
C14	0.0171 (7)	0.0176 (7)	0.0146 (7)	−0.0062 (6)	0.0020 (5)	−0.0077 (6)
C15	0.0163 (7)	0.0185 (7)	0.0140 (7)	−0.0073 (6)	0.0011 (5)	−0.0046 (6)
C16	0.0263 (9)	0.0183 (8)	0.0283 (10)	−0.0045 (7)	−0.0025 (7)	−0.0070 (7)
C17	0.0405 (11)	0.0222 (9)	0.0298 (11)	−0.0139 (8)	−0.0113 (9)	0.0036 (8)
C18	0.0395 (11)	0.0444 (12)	0.0150 (9)	−0.0294 (10)	0.0002 (7)	−0.0021 (8)
C19	0.0257 (9)	0.0476 (12)	0.0210 (9)	−0.0191 (8)	0.0087 (7)	−0.0181 (9)
C20	0.0169 (7)	0.0270 (9)	0.0192 (8)	−0.0065 (6)	0.0025 (6)	−0.0107 (7)
C21	0.0262 (9)	0.0348 (10)	0.0230 (9)	−0.0188 (8)	0.0058 (7)	−0.0134 (8)
O2	0.0213 (7)	0.0337 (9)	0.0305 (9)	−0.0104 (6)	−0.0044 (6)	−0.0144 (7)
C11	0.0200 (10)	0.0367 (13)	0.0351 (14)	−0.0058 (9)	−0.0053 (9)	−0.0179 (11)
C12	0.0316 (12)	0.0354 (13)	0.0353 (13)	−0.0111 (10)	−0.0007 (10)	−0.0187 (11)
O2A	0.0213 (7)	0.0337 (9)	0.0305 (9)	−0.0104 (6)	−0.0044 (6)	−0.0144 (7)
C11A	0.016 (5)	0.032 (6)	0.006 (5)	−0.007 (4)	−0.003 (4)	−0.007 (4)
C12A	0.037 (8)	0.059 (9)	0.038 (8)	−0.026 (7)	−0.014 (6)	−0.003 (6)

### Geometric parameters (Å, °)

**Table:**

Br1—C5	1.8982 (17)	C15—C20	1.383 (2)
S1—C14	1.6867 (16)	C16—C17	1.392 (3)
F1—C2	1.353 (2)	C16—H16	0.95
O1—C9	1.201 (2)	C17—C18	1.386 (4)
N1—C14	1.325 (2)	C17—H17	0.95
N1—C7	1.463 (2)	C18—C19	1.377 (3)
N1—H1N1	0.85	C18—H18	0.95
N2—C14	1.378 (2)	C19—C20	1.394 (3)
N2—C13	1.412 (2)	C19—H19	0.95
N2—C15	1.450 (2)	C20—H20	0.95
C1—C2	1.371 (2)	C21—H21A	0.98
C1—C6	1.424 (3)	C21—H21B	0.98
C1—H1	0.95	C21—H21C	0.98
C2—C3	1.377 (3)	O2—C11	1.459 (3)
C3—C4	1.380 (3)	C11—C12	1.499 (3)
C3—H3	0.95	C11—H11A	0.99
C4—C5	1.388 (2)	C11—H11B	0.99
C4—H4	0.95	C12—H12A	0.98
C5—C6	1.390 (2)	C12—H12B	0.98
C6—C7	1.537 (2)	C12—H12C	0.98
C7—C8	1.510 (2)	O2A—C11A	1.431 (12)
C7—H7	1.00	C11A—C12A	1.501 (15)
C8—C13	1.349 (2)	C11A—H11C	0.99
C8—C9	1.481 (2)	C11A—H11D	0.99
C9—O2A	1.359 (3)	C12A—H12D	0.98
C9—O2	1.361 (2)	C12A—H12E	0.98
C13—C21	1.505 (2)	C12A—H12F	0.98
C15—C16	1.382 (2)		
			
C14—N1—C7	127.83 (14)	C15—C16—C17	119.04 (18)
C14—N1—H1N1	113.1	C15—C16—H16	120.5
C7—N1—H1N1	118.5	C17—C16—H16	120.5
C14—N2—C13	121.82 (14)	C18—C17—C16	119.8 (2)
C14—N2—C15	118.93 (13)	C18—C17—H17	120.1
C13—N2—C15	118.34 (13)	C16—C17—H17	120.1
C2—C1—C6	117.93 (17)	C19—C18—C17	120.74 (18)
C2—C1—H1	121.0	C19—C18—H18	119.6
C6—C1—H1	121.0	C17—C18—H18	119.6
F1—C2—C1	117.18 (17)	C18—C19—C20	119.90 (19)
F1—C2—C3	118.70 (16)	C18—C19—H19	120.1
C1—C2—C3	124.11 (18)	C20—C19—H19	120.1
C2—C3—C4	117.87 (16)	C15—C20—C19	119.04 (18)
C2—C3—H3	121.1	C15—C20—H20	120.5
C4—C3—H3	121.1	C19—C20—H20	120.5
C3—C4—C5	120.24 (17)	C13—C21—H21A	109.5
C3—C4—H4	119.9	C13—C21—H21B	109.5
C5—C4—H4	119.9	H21A—C21—H21B	109.5
C4—C5—C6	121.62 (16)	C13—C21—H21C	109.5
C4—C5—Br1	116.51 (13)	H21A—C21—H21C	109.5
C6—C5—Br1	121.87 (13)	H21B—C21—H21C	109.5
C5—C6—C1	118.20 (15)	C9—O2—C11	116.68 (18)
C5—C6—C7	123.85 (15)	O2—C11—C12	110.62 (19)
C1—C6—C7	117.90 (15)	O2—C11—H11A	109.5
N1—C7—C8	109.92 (14)	C12—C11—H11A	109.5
N1—C7—C6	107.99 (13)	O2—C11—H11B	109.5
C8—C7—C6	112.44 (13)	C12—C11—H11B	109.5
N1—C7—H7	108.8	H11A—C11—H11B	108.1
C8—C7—H7	108.8	C11—C12—H12A	109.5
C6—C7—H7	108.8	C11—C12—H12B	109.5
C13—C8—C9	126.36 (16)	H12A—C12—H12B	109.5
C13—C8—C7	121.87 (14)	C11—C12—H12C	109.5
C9—C8—C7	111.77 (15)	H12A—C12—H12C	109.5
O1—C9—O2A	107.4 (5)	H12B—C12—H12C	109.5
O1—C9—O2	123.54 (17)	C9—O2A—C11A	111.1 (8)
O1—C9—C8	121.38 (17)	O2A—C11A—C12A	99.2 (10)
O2A—C9—C8	124.7 (5)	O2A—C11A—H11C	111.9
O2—C9—C8	114.94 (16)	C12A—C11A—H11C	111.9
C8—C13—N2	120.36 (15)	O2A—C11A—H11D	111.9
C8—C13—C21	125.54 (15)	C12A—C11A—H11D	111.9
N2—C13—C21	114.07 (15)	H11C—C11A—H11D	109.6
N1—C14—N2	117.58 (14)	C11A—C12A—H12D	109.5
N1—C14—S1	121.21 (12)	C11A—C12A—H12E	109.5
N2—C14—S1	121.21 (12)	H12D—C12A—H12E	109.5
C16—C15—C20	121.44 (16)	C11A—C12A—H12F	109.5
C16—C15—N2	120.12 (15)	H12D—C12A—H12F	109.5
C20—C15—N2	118.31 (15)	H12E—C12A—H12F	109.5

### Hydrogen-bond geometry (Å, °)

**Table:**

*D*—H···*A*	*D*—H	H···*A*	*D*···*A*	*D*—H···*A*
N1—H1N1···S1^i^	0.85	2.51	3.327 (2)	162
C1—H1···F1^ii^	0.95	2.52	3.370 (2)	148
C7—H7···Br1	1.00	2.69	3.265 (2)	117
C20—H20···O1^iii^	0.95	2.44	3.368 (3)	164
C21—H21A···O2	0.98	2.11	2.737 (3)	120
C21—H21B···Br1^iii^	0.98	2.91	3.886 (2)	171

Symmetry codes: (i) −*x*+2, −*y*+1, −*z*; (ii) −*x*+2, −*y*, −*z*; (iii) −*x*+1, −*y*+1, −*z*.
